# We are not so special

**DOI:** 10.7554/eLife.38726

**Published:** 2018-07-03

**Authors:** Zachary R Lewis, Casey W Dunn

**Affiliations:** Department of Ecology and Evolutionary BiologyYale UniversityNew HavenUnited States

**Keywords:** choanoflagellates, Urmetazoan, innate immunity, ancestral gene content, transcriptome, Other

## Abstract

New sequence data from choanoflagellates improves our understanding of the genetic changes that occurred along the branch of the evolutionary tree that gave rise to animals.

**Related research article** Richter DJ, Fozouni P, Eisen M, King N. 2018. Gene family innovation, conservation and loss on the animal stem lineage. *eLife* **7**:e34226. doi: 10.7554/eLife.34226

The most recent common ancestor of animals lived more than 600 million years ago, so we cannot sequence its genome. Nevertheless, we can identify a minimal set of gene families that were present in this long-dead ancestor by comparing genomic data across animals and their closest relatives. In addition to being interesting in its own right, this helps us identify which genes were gained and lost before the origin of animals and, likewise, which genes were gained and lost as animals diversified.

The challenge, though, is that there are strong sampling biases that can compromise these analyses. Genome sequencing has focused on species that are medically relevant, experimentally tractable, and easy to sequence (del Campo et al., 2014). Left unaddressed, these biases can frustrate efforts to reconstruct the genomes of our ancient ancestors. Take, for example, the simple case of three groups of organisms called O, C and M, and a gene that originated along the branch that gave rise to C and M (Figure 1A). If more sequencing effort has been invested in group M than in group C, the gene is more likely to be found in group M than in group C. And if the gene is found in M but not in C, even though it is present in both, then it will appear that the gene is specific to group M and younger than it actually is.

**Figure 1.**
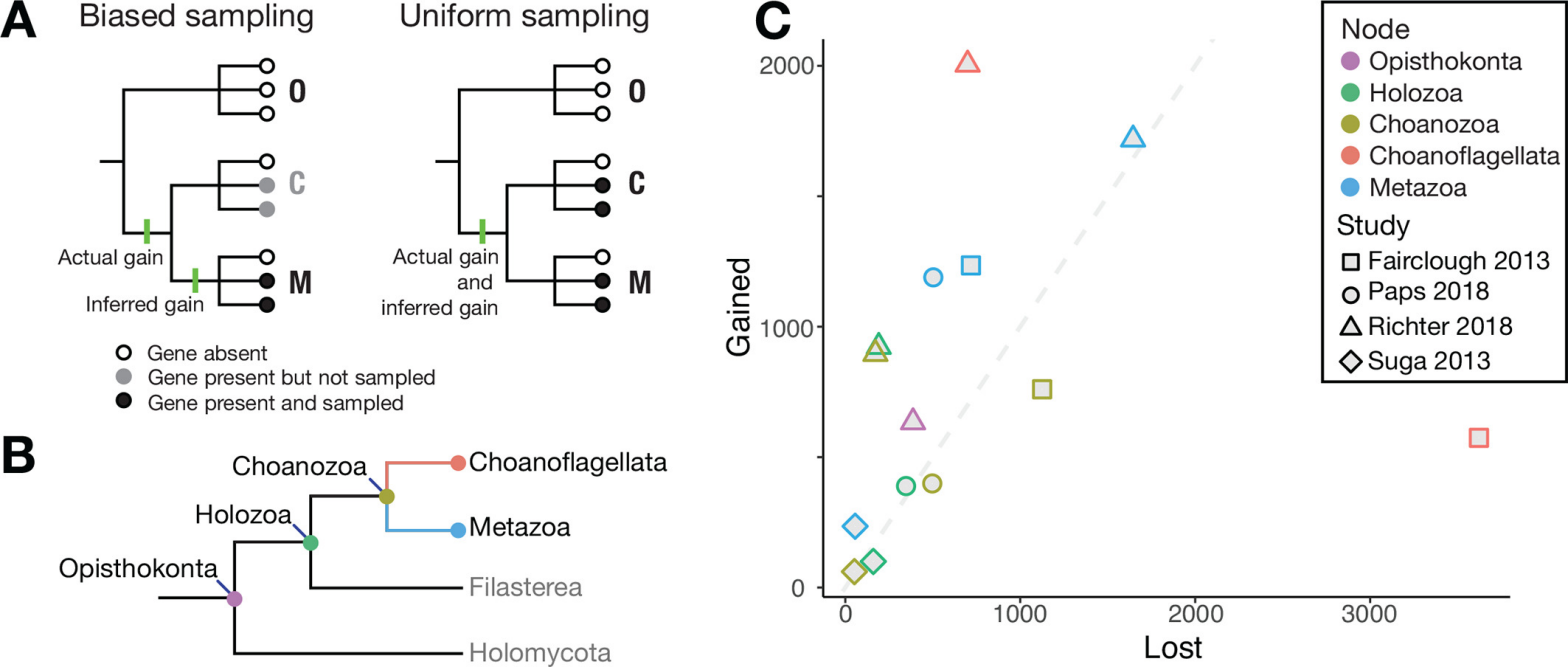
Genes lost and gained. (**A**) Example of biased sampling (left): although a gene was gained (first green line) before group C and group M diverged, biased sampling means that it is only detected in group M, which leads to the incorrect inference (second green line) that the gene arose after the groups diverged. With uniform sampling (right), the gene gain is correctly inferred (third green line). Groups C, M and O could be Choanoflagellata, Metazoa and Outgroups. (**B**) Cladogram showing the evolutionary relationships of the clades in question, with the Choanoflagellata stem shown in red and the Metazoa stem shown in blue. Choanozoa refers to the clade Choanoflagellata + Metazoa (Brunet and King, 2017). (**C**) The number of gene groups gained (y-axis) plotted against the number of gene groups lost (x-axis) along various branches leading to the nodes shown in panel B, based on the data in four studies (Fairclough et al., 2013; Paps and Holland, 2018; Richter et al., 2018; Suga et al., 2013). The gray dashed line indicates equal gene group gain and loss. Note that the four studies use different methodologies to define groupings of genes. Data and analyses are available at https://github.com/dunnlab/gene_inventory_2018 (Lewis and Dunn, 2018; copy archived at https://github.com/elifesciences-publications/gene_inventory_2018).

Now, in eLife, Daniel Richter, Parinaz Fozouni, Michael Eisen and Nicole King report their work to reduce sequencing bias by sampling many more genes in the sister group to animals, the choanoflagellates (Richter et al., 2018). They generated transcriptomic data for 19 species of choanoflagellates and analyzed them in combination with previously published metazoan (animal), choanoflagellate and other eukaryote genomes. In addition to presenting new data, Richter et al. – who are based at UC Berkeley, UCSF, the Gladstone Institutes and Station Biologique de Roscoff – applied new probabilistic methods to minimize the chance that a gene family would be predicted to be present in a taxonomic group based on the spurious assignment of unrelated genes to the same family.

In related work at the universities of Essex and Oxford, Jordi Paps and Peter Holland have reported an interesting analysis of gene gain and loss in early animal evolution (Paps and Holland, 2018). The studies agree on some key points. Both recovered a relatively large number of gene family gains along the ‘animal stem’ (the branch of the evolutionary tree that uniquely gives rise to animals; shown in blue in Figure 1B). However, while Paps and Holland estimate that the number of gains was much higher than the number of losses, which they interpreted as evidence for an accelerated expansion of gene families along the Metazoa stem, Richter et al. estimate approximately equal numbers of gains and losses (Figure 1C). This means that Richter et al. find evidence for accelerated churn of gene families along the Metazoa stem, not a burst of expansion. This incongruence is likely related to Paps and Holland analyzing two choanoflagellate species, compared to the 21 analyzed by Richter et al.

Another difference is that Paps and Holland did not estimate gene gain and loss along the Choanoflagellata stem, whereas Richter et al. did. This revealed more gene family gain and less gene family loss along the Choanoflagellata stem than along the Metazoa stem (Figure 1C). So, Richter et al. do find a burst of gene family expansion, but in Choanoflagellata rather than Metazoa. It will be critical to further test the findings of both studies with improved sampling of other closely related groups, which could change how the gains and losses are apportioned to these two stems.

The results presented by Richter et al. agree in important ways with other recent work (King et al., 2008; Suga et al., 2013). These analyses reveal that the genetic changes on the Metazoa stem included the evolution of new intercellular signaling pathways (Fairclough et al., 2013) and the integration of new ligands and receptors into intracellular pathways that were already present (such as the Hippo pathway; Sebé-Pedrós et al., 2012). Other changes included the expansion of a core set of transcription factors (de Mendoza et al., 2013), and increased cis-regulatory complexity (Sebé-Pedrós et al., 2016).

Comparative gene content analyses refine our understanding of what makes metazoans unique, and in the process we are learning about the underappreciated biology of our close non-metazoan relatives (Sebé-Pedrós et al., 2017). For instance, Richter et al. identified homologs of Toll-like receptors in most choanoflagellates. These genes were thought to be an animal-specific innovation for innate immunity. Future research could investigate if these genes have immune-like roles in non-animals.

It is impossible to know how special animals really are without also knowing something about our closest relatives. The more we learn about these relatives, the less special we seem to be.

## References

[bib1] Brunet T, King N (2017). The origin of animal multicellularity and cell differentiation. Developmental Cell.

[bib2] de Mendoza A, Sebé-Pedrós A, Šestak MS, Matejcic M, Torruella G, Domazet-Loso T, Ruiz-Trillo I (2013). Transcription factor evolution in eukaryotes and the assembly of the regulatory toolkit in multicellular lineages. PNAS.

[bib3] del Campo J, Sieracki ME, Molestina R, Keeling P, Massana R, Ruiz-Trillo I (2014). The others: our biased perspective of eukaryotic genomes. Trends in Ecology & Evolution.

[bib4] Fairclough SR, Chen Z, Kramer E, Zeng Q, Young S, Robertson HM, Begovic E, Richter DJ, Russ C, Westbrook MJ, Manning G, Lang BF, Haas B, Nusbaum C, King N (2013). Premetazoan genome evolution and the regulation of cell differentiation in the choanoflagellate *Salpingoeca rosetta*. Genome Biology.

[bib5] King N, Westbrook MJ, Young SL, Kuo A, Abedin M, Chapman J, Fairclough S, Hellsten U, Isogai Y, Letunic I, Marr M, Pincus D, Putnam N, Rokas A, Wright KJ, Zuzow R, Dirks W, Good M, Goodstein D, Lemons D, Li W, Lyons JB, Morris A, Nichols S, Richter DJ, Salamov A, Sequencing JG, Bork P, Lim WA, Manning G, Miller WT, McGinnis W, Shapiro H, Tjian R, Grigoriev IV, Rokhsar D (2008). The genome of the choanoflagellate *Monosiga brevicollis* and the origin of metazoans. Nature.

[bib6] Lewis ZR, Dunn CW (2018). Github.

[bib7] Paps J, Holland PWH (2018). Reconstruction of the ancestral metazoan genome reveals an increase in genomic novelty. Nature Communications.

[bib8] Richter DJ, Fozouni P, Eisen M, King N (2018). Gene family innovation, conservation and loss on the animal stem lineage. eLife.

[bib9] Sebé-Pedrós A, Ballaré C, Parra-Acero H, Chiva C, Tena JJ, Sabidó E, Gómez-Skarmeta JL, Di Croce L, Ruiz-Trillo I (2016). The dynamic regulatory genome of *Capsaspora* and the origin of animal multicellularity. Cell.

[bib10] Sebé-Pedrós A, Degnan BM, Ruiz-Trillo I (2017). The origin of Metazoa: a unicellular perspective. Nature Reviews Genetics.

[bib11] Sebé-Pedrós A, Zheng Y, Ruiz-Trillo I, Pan D (2012). Premetazoan origin of the Hippo signaling pathway. Cell Reports.

[bib12] Suga H, Chen Z, de Mendoza A, Sebé-Pedrós A, Brown MW, Kramer E, Carr M, Kerner P, Vervoort M, Sánchez-Pons N, Torruella G, Derelle R, Manning G, Lang BF, Russ C, Haas BJ, Roger AJ, Nusbaum C, Ruiz-Trillo I (2013). The *Capsaspora* genome reveals a complex unicellular prehistory of animals. Nature Communications.

